# Verbascoside Protects Pancreatic β-Cells against ER-Stress

**DOI:** 10.3390/biomedicines8120582

**Published:** 2020-12-08

**Authors:** Alessandra Galli, Paola Marciani, Algerta Marku, Silvia Ghislanzoni, Federico Bertuzzi, Raffaella Rossi, Alessia Di Giancamillo, Michela Castagna, Carla Perego

**Affiliations:** 1 Department of Pharmacological and Biomolecular Sciences, Università degli Studi di Milano, 20134 Milan, Italy; alessandra.galli1@unimi.it (A.G.); paola.marciani@unimi.it (P.M.); Algerta.marku@unimi.it (A.M.); silvia.ghislanzoni1@studenti.unimi.it (S.G.); michela.castagna@unimi.it (M.C.); 2 Diabetology Unit, Niguarda Hospital, 20162 Milan, Italy; federico.bertuzzi@ospedaleniguarda.it; 3 Department of Veterinary Medicine, Università degli Studi di Milano, 26900 Lodi, Italy; raffaella.rossi@unimi.it (R.R.); alessia.digiancamillo@unimi.it (A.D.G.)

**Keywords:** verbascoside, polyphenols, insulin-producing cells, diabetes, UPR, oxidative stress, ER-stress, PERK, anti-inflammatory, mitochondria

## Abstract

Substantial epidemiological evidence indicates that a diet rich in polyphenols protects against developing type 2 diabetes. The phenylethanoid glycoside verbascoside/acteoside, a widespread polyphenolic plant compound, has several biological properties including strong antioxidant, anti-inflammatory and neuroprotective activities. The aim of this research was to test the possible effects of verbascoside on pancreatic β-cells, a target never tested before. Mouse and human β-cells were incubated with verbascoside (0.8–16 µM) for up to five days and a combination of biochemical and imaging techniques were used to assess the β-cell survival and function under normal or endoplasmic reticulum (ER)-stress inducing conditions. We found a dose-dependent protective effect of verbascoside against oxidative stress in clonal and human β-cells. Mechanistic studies revealed that the polyphenol protects β-cells against ER-stress mediated dysfunctions, modulating the activation of the protein kinase RNA-like endoplasmic reticulum kinase (PERK) branch of the unfolded protein response and promoting mitochondrial dynamics. As a result, increased viability, mitochondrial function and insulin content were detected in these cells. These studies provide the evidence that verbascoside boosts the ability of β-cells to cope with ER-stress, an important contributor of β-cell dysfunction and failure in diabetic conditions and support the therapeutic potential of verbascoside in diabetes.

## 1. Introduction

Diabetes is a chronic disorder affecting hundreds of million people [1]. Different etiology characterizes Type 1 (T1D) and Type 2 diabetes (T2D) both featuring lack of insulin [2]. Insulin regulates plasma glucose concentration stimulating glucose uptake in muscle and fat cells and modulating liver glucose metabolism. Nutrient availability, hormones, and neural inputs regulate pancreatic insulin secretion and maintain blood glucose concentrations within a physiological range [3,4,5]. Therefore, β-cell dysfunction leads to diabetes characterized by fasting hyperglycemia. 

T2D is a progressive condition in which insulin resistance and β-cells misfunctions are linked together and recent evidence rose interest in the critical primary role of β-cells to the hyperglycemic status [6,7]. In non-diabetic obese subjects β-cells compensate insulin resistance with increased secretion of the hormone. However, in some subjects, as their conditions deteriorate, insulinemia drops and glucose level climbs to hyperglycemia [8,9]. 

Glucose is the most important modulator of β-cell functions. Glucose stimulation affects regulation of genes and expression of proteins involved in many cell functions such as glycolysis, insulin synthesis and secretion [10]. Glucose-induced insulin secretion relies on oxidative metabolism to produce adenosine triphosphate (ATP) and a low level of reactive oxygen species (ROS) is physiologically produced. However, β-cells are not very well equipped with scavenger enzymes and this weakness makes them very susceptible to oxidative stress [11,12]. As oxidative stress climbs β-cells insulin production declines, and β-cells produce cytokines that ignite cell damage through inflammation and apoptosis [13]. Interestingly, although the reduced β-cells mass has been previously attributed to cell death, recent studies suggest a major role of cell dedifferentiation [14,15]. It has become clear that several stages drive to T2D. Metabolic and oxidative insults cause endoplasmic reticulum (ER) stress which leads to decline of insulin synthesis and secretion, inflammation processes follow the release of cytokines and β-cells loss may occur through apoptosis and dedifferentiation. This knowledge is fundamental in order to develop appropriate strategies of treatment, since oxidative stress and inflammation can be counteracted, and the plasticity of pancreatic cells allows the possibility to revert stem cells to β-cells as proved in mice [16].

Recently, the vulnerability of β-cells to oxidative stress has successfully prompted the use of dietary antioxidants to prevent diabetes [17]. Among the most interesting antioxidant food, olive oil is known for its beneficial properties since the ancient Greeks. Olive oil contains good amount of mono-unsaturated fatty acids (MUFA) and several polyphenols such as tyrosol, hydroxytyrosol, oleuropein, and verbascoside. 

Verbascoside, also known as acteoside, is a phenylethanoid glycoside extracted from *Olea europea*, plants of the *Verbascum* species, and 23 other plant families [18,19,20]. It can also be obtained from olive oil by-products (it is enriched in olive-mill wastewater derived from olive fruit processing) or be produced by metabolic engineering and synthetic biology approaches [21].

No data have been reported about verbascoside bioavailability in humans, yet. Studies conducted on mice, SKBR3 and Caco-2 cells suggest that it may be feasible for non-metabolized verbascoside to cross the intestinal barrier, circulate in blood plasma, and exert antioxidant effects [20,22,23]. Unlike most of plant polyphenols, verbascoside mainly acts on cells through the modulation of gene transcription of a variety of enzymes and regulatory factors, with antioxidant and anti-inflammatory effects [24,25,26,27,28,29,30].

Although many beneficial effects of verbascoside for human health are known, there are no data about its effect on pancreatic β-cells. Since oxidative stress and inflammation are at the basis of T2D pathogenesis, we investigated whether verbascoside treatment might improve β-cells viability and function in ER-stress inducing conditions and we characterized the molecular mechanisms of its action. Our data reveal that verbascoside prevents β-cells oxidative stress and inflammation modulating the activation of the unfolded protein response and promoting mitochondrial dynamics, thus resulting in increased β-cell viability and insulin content.

## 2. Materials and Methods

### 2.1. Cells Culture and Materials 

Mouse βtc3 cells (kindly provided by Prof. Hanahan—Department of Biochemistry and Biophysics, University of California, San Francisco, CA [31]) were cultured in RPMI 1640 medium (Euroclone S.p.A, ECB900, Pero MI, Italy) supplemented with 10% (*v*/*v*) heat-inactivated fetal calf serum (Euroclone S.p.A, ECS0180L, Pero MI, Italy), 1% (*v*/*v*) penicillin-streptomycin (EuroClone S.p.A., ECB3001D, Pero MI, Italy) and 1% (*v*/*v*) L-glutamine (EuroClone S.p.A., ECB300D, Pero MI, Italy). Human islets of Langerhans were isolated in Milan (Niguarda Ca’ Granda) from cadaveric multiorgan donors according to the procedure described by Ricordi et al. [32]; they were cultured in RPMI culture medium containing 5.5 mmol/L glucose, 10% heat-inactivated fetal bovine serum, 0.7 mM Glutamine, 50 units/mL penicillin and 50 μg/mL streptomycin (EuroClone, S.p.A., Pero MI, Italy). Four different islets preparation were used, islets purity was 80 ± 10%. Islet isolation and islet studies were approved by the Ethics Committee of the Niguarda Ca’ Granda hospital in Milan (11.12.2009). 

βtc3 cells were treated with 0.8, 1.6, and 16 μM verbascoside (Carbosynth, OV08034, Compton, UK), caffeic acid and hydroxytyrosol (kind gift of Prof. Dell’Agli, Department of Pharmacological and Biomolecular Sciences, Università degli Studi di Milano, Milan, Italy) in complete RPMI medium for 5 days, while human islets with 16 μM verbascoside. Methanol/ethanol treated cells were used as controls. In order to induce oxidative stress, cells were treated with H_2_O_2_ (Sigma Aldrich, H1009, St. Louis, MO, USA) 500 μM in complete RPMI medium for 20 min before analysis, while treatment with tunicamycin 2 μg/mL (T7765, Sigma Aldrich) for 7 h was performed to induce ER stress. 

### 2.2. Detection of Cell Viability by MTT Test 

Cells were plated in 96 multi-well plates, and five days after verbascoside treatment they were incubated with 0.5 mg/mL MTT (3-(4,5-dimethyltiazol-2-yl)-2,5-diphenyltetrazolium bromide) (Sigma-Aldrich, M5655, St. Louis, MO, USA) for 4 h in humidified atmosphere containing 5% of CO_2_ at 37 °C. After incubation, cells were gently resuspended in 100 μL DMSO (Euro-Clone S.p.A., BK12611S, Pero MI, Italy) and the absorbance at 540 nm was detected with a microplate reader (Benchmark, microplate reader, Bio-Rad Laboratories, Hercules CA, USA) [33]. Experiments were performed in triplicate and data were expressed as fold increase over control samples.

### 2.3. Detection of Cell Death by Flow Cytometry 

Five days after incubation with 16 µM verbascoside, the β-cells were detached by 7 min incubation with trypsin/EDTA, collected and centrifuged; the pellet was gently resuspended in phosphate buffer saline low salts. Cells were stained with Muse^TM^ count and viability reagent (Millipore, MCH100102, Burlington MA, USA) following the manufacturer’s protocol and analyzed through flow cytometry. Experiments were performed in triplicate and data were expressed as percentage of dead cells over the total.

### 2.4. ROS Generation

Intracellular ROS were evaluated with DCFDA (2′,7′-dichlorofuorescein diacetate) (Sigma Aldrich, D6883, St. Louis, MO, USA), a membrane permeable probe that becomes fluorescent when tied to ROS [34]. βtc3 cells were pre-loaded with 15 μM DCFDA in Krebs–Ringer buffer (125 mM NaCl, 5 mM KCl, 1.2 mM MgSO_4_, 1.2 mM KH_2_PO_4_, 25 mM HEPES-NaOH pH 7.4 and 2 mM CaCl_2_) supplemented with 11 mM glucose for 1 h at 37 °C. The ROS content was detected for 30 min both in basal and stress conditions with a microplate reader (485/528 nm Ex/Em) (TECAN Infinite^®^ F500, Tecan Group Ltd. Männedorf, Switzerland). Mean values and standard deviations were based on three independent experiments.

### 2.5. Western Blotting

βtc3 cells were collected and solubilized in RIPA buffer (150 mM NaCl, 50 mM Tris HCl pH 7.6, 1 mM EDTA, 1% TERGITOL™ NP40, 0.5% deoxycholate) added with aprotinin (Sigma Aldrich, A4529, St. Louis, MO, USA), PMSF (Sigma Aldrich, 10837091001, St. Louis, MO, USA) and Roche inhibitors (Sigma Aldrich, 5892953001, St. Louis, MO, USA) for 40 min at 4 °C. Protein concentration was determined by Bradford assay [35] by using Bradford Reagent (Sigma Aldrich, B6916, St. Louis, MO, USA), 30 μg of proteins were resolved by 10% SDS-PAGE and transferred onto nitrocellulose membranes (Millipore, Burlington MA, USA). Primary antibodies were applied for 2 h in blocking buffer with 5% non-fat milk or 5% BSA solutions; the following primary antibodies were used: mouse anti-β-actin (Novus International Inc., NB600501, St. Louis, MO, USA), mouse anti-acrolein (Abcam, ab48501, Cambridge, UK), mouse anti-BIP (kind gift of Prof. Borgese Nica, Institute of Neuroscience, CNR, Milan, Italy), rabbit anti-HNE (α-diagnostic International Inc., HNE11S, San Antonio, TX, USA), mouse anti-HSP70 (Enzo Life Sciences Inc., C92F3A-5, Farmingdale, NY, USA), rabbit anti-phospho-IκBα (Ser32) (Cell Signaling Technology Inc., 3033, Danvers, MA, USA), mouse anti-IκBα (Cell Signaling Technology Inc., 4814, Danvers, MA, USA), rabbit anti-phospho-NFκB p65 (Ser 536) (Cell Signaling Technology Inc., 3033, Danvers, MA, USA), rabbit anti-NFkB p65 (Cell Signaling Technology Inc., 8242, Danvers, MA, USA), sheep anti-SOD1 (Merck, KGaA, Darmstadt, Germania), rabbit anti-PERK (Cell Signaling Technology Inc., 3192, Danvers, MA, USA), rabbit anti-eIF2α (Cell Signaling Technology Inc., 5324, Danvers, MA, USA) and rabbit anti-P-eIF2α (Ser 51) (Cell Signaling Technology Inc., 3597, Danvers, MA, USA). The secondary antibodies HRP-conjugated (Dako Agilent, Santa Clara, CA, USA) were used at 1:5000 dilution. Proteins were detected by using the ECL detection system (Euro-Clone S.p.A., Pero MI, Italy) by using Odyssey Fc Image system (LI-COR Biotechnology GmbH, Bad Homburg, Germany) and band density was quantified by Image Studio™ Lite software (LI-COR Biosciences, Lincoln NE, USA) [36]. Experiments were performed in triplicate and data were expressed as fold increase over control samples.

### 2.6. Mitochondrial Membrane Potential

βtc3 cells and human islets of Langerhans were incubated with 100 nM MitoSpy™ Orange CMTMRos (BioLegend, 424803, Campoverde Srl, Milan, Italy) or 100 μM MitoSpy™ Green FM (BioLegend, 424805, Campoverde Srl, Milan, Italy) for 30 min at 37 °C; fluorescence intensities were detected with the microplate reader TECAN Infinite^®^ F500 (551/576 nm Ex/Em for MitoSpy™ Orange CMTMRos; 490/516 nm Ex/Em for MitoSpy™ Green) (TECAN Infinite^®^ F500, Tecan Group Ltd. Männedorf, Switzerland). Cells were incubated with 500 μM H_2_O_2_ for 20 min and fluorescence intensity was detected as previously described. Mean values and standard deviations were based on three different experiments.

### 2.7. Mitochondrial Morphology and Dynamics

βtc3 cells were pre-loaded with 100 nM MitoSpy™ Orange CMTMRos (BioLegend, 424803, Campoverde Srl, Milan, Italy) in 11 mM glucose Krebs–Ringer buffer at 37 °C for 30 min. Samples were positioned in an imaging chamber and random fields were imaged by using the rhodan filter of the Axio Observer Z1 microscope (Zeiss, Oberkochen Germany). To evaluate mitochondrial morphology, the following parameters were analyzed by using the ImageJ particle analyzer software: area (μm^2^), circularity (4πArea^2^/Perimeter^2^) and Feret’s maximum diameter (μm) [37]. 

For time-lapse experiments, single-cell imaging was carried out at 1 frame per second for 30 s under control or oxidative stress conditions. To measure the mitochondrial cumulative distance (µm^2^), images were first corrected for photo bleaching, then videos were analyzed by using an existing Image-Pro Plus Plug-in (object tracking) (Media Cybernetics, Rockville, MD, USA). Up to twelve cells were imaged in three independent experiments and data were presented as mean values and standard deviations.

### 2.8. Insulin Secretion 

Human isolated islets of Langerhans were seeded in 96-well plate at a density of 20 islets per well and, after 5 days of treatment, insulin content and secretion were measured in basal (3.3 mM glucose) and stimulated (16.7 mM glucose) conditions by means of an ELISA immunoassay (Mercodia, 10-1113-01, Uppsala, Sweden).

### 2.9. Statistical Analyses

All statistical analyses were performed with GraphPad Prism 8.0 on independent biological replicates. Means between two groups were evaluated by using the two-tailed Student’s *t*-test and a *p*-value < 0.05 was taken as evidence of statistical significance. Means among three or more groups were compared by analysis of variance (ANOVA), followed by multiple post-hoc (Tukey’s) comparison test. The statistical test used, exact P values and the number of replica (n) are indicated in the individual figure legends. Error bars in the figures display the mean ± S.D. or the mean ± S.E., as indicated.

## 3. Results

### 3.1. Verbascoside Improves β-Cells Viability

**Figure 1:**
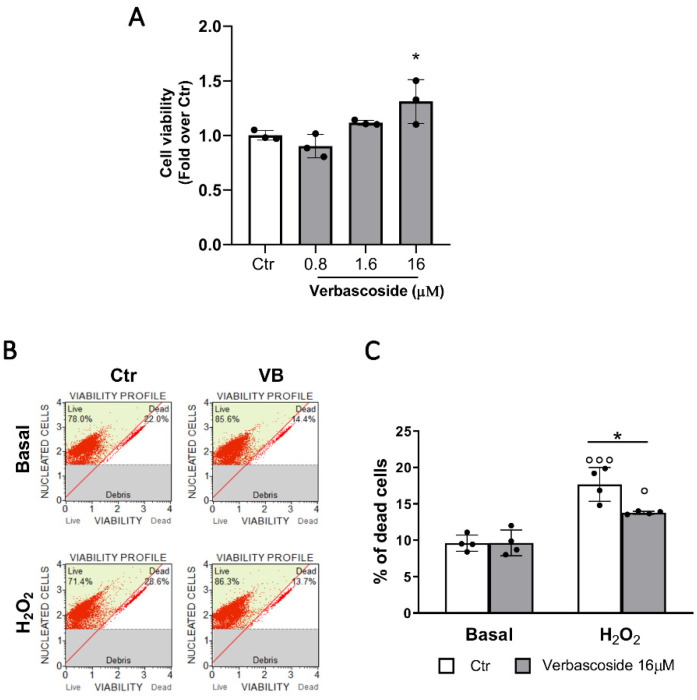
Verbascoside improves β-cell viability. Mouse βtc3 cells were treated with 0.8, 1.6, and 16 μM verbascoside (VB) for 5 days and methanol treated cells were used as controls. (**A**) MTT test. Data of three independent experiments (mean values ± SD) are expressed as fold change over control. (One-way ANOVA, post-hoc Tukey’s test * *p =* 0.046 VB vs. Ctr). (**B**) Representative images of flow cytometry experiments. βtc3 cells were trypsinized, labelled with Muse^TM^ count and viability reagent, and analyzed through flow cytometry. Plot organization. Lower panel: cellular debris; upper panel: percentage of live (right part) and dead (left part) cells. (**C**) Quantification of β-cell death by flow cytometry. Data (mean values ± SD) are expressed as percentage of dead cells over total cells; experiments were performed in quadruplicate (two-way ANOVA, post-hoc Tukey’s test. ° *p* = 0.012, °°° *p* < 0.0001 H_2_O_2_ vs. Basal; * *p* = 0.018 VB vs. Ctr).

Since there were no data on verbascoside effect on β-cells, we performed MTT test to evaluate cell viability and as shown in Figure 1A, a positive dose-dependent trend was observed. Hence, for further studies, we selected the 16 μM concentration that statistically was proven effective to enhance β-cells viability. 

By flow cytometry we studied the impact of five days incubation of verbascoside on β-cell survival in basal conditions and after 20 min of pre-treatment with 500 μM H_2_O_2_. In the basal condition, verbascoside did not affect cell survival, suggesting that the increased β-cell viability observed in the MTT assay may be due to improved mitochondrial activity. Whereas, after H_2_O_2_ exposure, 16 μM verbascoside pre-treatment significantly reduced oxidative stress-induced β-cell death (Figure 1B,C).

Verbascoside is a complex molecule that can be modified by hydrolyzing enzymes [18]. To test whether verbascoside metabolites could be responsible for the observed protective effect, βtc3 cells were treated with hydroxytyrosol and caffeic acids (0.8 and 16 μM) for 5 days. Both metabolites did not enhance cell viability, on the contrary a cytotoxic effect, more relevant after H_2_O_2_ exposure, was detected for the 16 µM concentration (Figure S1). This finding proves that verbascoside and not its metabolites exerts the protective effect to H_2_O_2_ treatment. 

### 3.2. Verbascoside Modulates the Redox Homeostasis and Exerts an Anti-Inflammatory Effect in β-Cells

**Figure 2:**
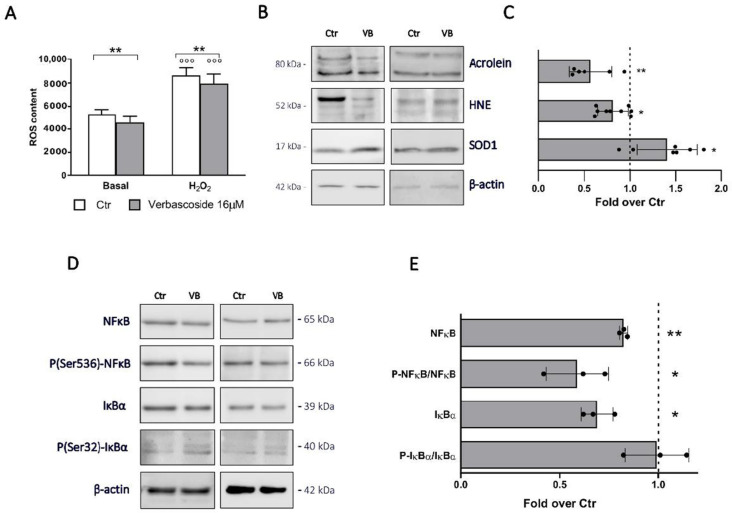
Verbascoside modulates redox homeostasis and inflammation in βtc3 cells. (**A**) ROS content. Intracellular ROS were monitored by DCFDA and quantified by fluorimetry (485/528 nm Ex/Em) under basal and oxidative stress (H_2_O_2_ 500 µM for 30 min) conditions. Data are expressed as mean ± SD of three independent experiments. (Two-way ANOVA, post-hoc Tukey’s test. °°° *p* < 0.0001 H_2_O_2_ vs. Basal; ** *p* = 0.0055 VB vs. Ctr). (**B**) Western blotting analysis of HNE, acrolein and SOD1 in cells treated with 16 μM verbascoside (VB) for 5 days (30 μg protein/sample). On the left, the molecular-weight size markers in kDa are reported. (**C**) Quantitative analysis of protein expression shows upregulation of SOD1 and reduction of HNE and acrolein in cells treated with 16 μM verbascoside. Data (mean values ± SD) are expressed as fold change over control (dashed line). (n = 6–9 independent experiments). (Student’s t-test * *p* < 0.05, ** *p* < 0.01 VB vs. Ctr). (**D**) Western blotting analysis of NFκB pathways selected proteins in cells treated with 16 μM verbascoside (VB) for 5 days (30 μg protein/sample). On the right, the molecular-weight size markers in kDa are reported. (**E**) Quantitative analysis of protein expression shows that verbascoside treatment downregulates the activation of the NFκB pathway. Data (mean values ± SD) are expressed as fold change over control (dashed line). (n = 3 independent experiments performed in triplicate), (Student’s t-test * *p* < 0.05, ** *p* < 0.01 VB vs. Ctr).

We first confirmed in βtc3 cells the anti-inflammatory and anti-oxidant effects of verbascoside observed in other cell types [24,29,34]. The verbascoside ROS scavenging activity was evaluated with βtc3 cells labelled with the ROS specific DCFDA (2′,7′-dichlorofuorescein diacetate) cellular permeable probe. As shown in Figure 2A, a significant decrease of ROS content was detected after verbascoside pre-treatment, both under basal and oxidative stress conditions. 

Western blot analyses revealed a significant decrease of oxidative stress markers acrolein and 4-hydroxynonenal (HNE) expression in verbascoside-treated cells. In addition, we found an increase of superoxide dismutase (SOD1) expression, suggesting that verbascoside exerts its antioxidant activity probably with two different mechanisms, directly as a ROS scavenger and indirectly by inducing the expression of antioxidant enzymes (Figure 2B,C).

The anti-inflammatory effect of verbascoside on β-cells was assessed by evaluating the activation of the NFκB pathway, the most important pro-inflammatory pathway in these cells [38]. By western blot analysis we found a reduced expression of inhibitor of nuclear factor kappa B (IκBα) and nuclear factor kappa-light-chain-enhancer (NFκB), and a significant decrease of NFκB phosphorylation in pre-treated cells, backing the hypothesis of the verbascoside effectiveness to reduce cellular inflammation (Figure 2D,E). 

### 3.3. Verbascoside Modulates the Unfolded Protein Response of β-Cells

We then analyzed in depth the molecular mechanism by which verbascoside exerts antioxidant and anti-inflammatory roles, focusing on the endoplasmic reticulum which is emerging as a key sensor of metabolic and stress signals in β-cells. Under stress conditions, the organelle mounts a homeostatic response, known as the unfolded protein response (UPR), aimed at recovering the ER function; however, the excessive activation of this pathway results in apoptosis [39,40]. 

**Figure 3:**
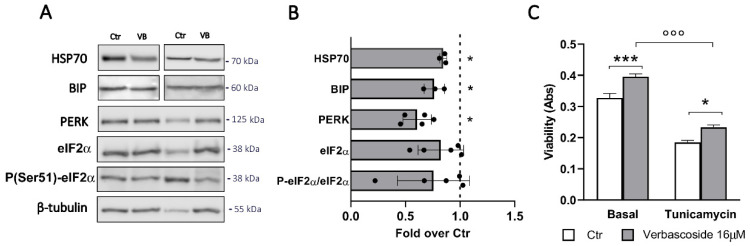
Verbascoside reduces ER stress. (**A**) Western blot analysis of ER stress markers in cells treated with 16 μM verbascoside (VB) for 5 days (30 μg protein/sample). On the right, the molecular-weight size markers in kDa are reported. (**B**) The quantitative analysis shows that verbascoside treatment reduces the expression of HSP70, BIP and PERK proteins. Data (mean values ± SD) are expressed as fold change over control (dashed line). (n = 3–5 independent experiments performed in triplicate) (Student’s t-test * *p* < 0.05 vs. Ctr). (**C**) Mouse βtc3 cells were incubated with 16 μM verbascoside (VB) for 5 days and ER stress was induced by 2 μg/mL tunicamycin treatment for 7 h. MTT test reveals a protective role of verbascoside against the tunicamycin-induced ER stress. Data are expressed as mean ± SD of three independent experiments (two-way ANOVA, post-hoc Tukey’s test. °°° *p* < 0.0001 tunicamycin vs. Basal; * *p* = 0.024 VB vs. Ctr; *** *p* = 0.0001).

Markers of increased ER stress are the upregulation of chaperones proteins and activation of the UPR response. Western blotting analysis of cells treated with verbascoside revealed decreased levels of the two chaperonins heat shock protein 70 (HSP70) and binding immunoglobulin protein (BIP) (Figure 3A,B). Furthermore, a significant reduction of protein kinase RNA-like ER kinase (PERK) expression and decreased phosphorylation of its downstream effector, the eukaryotic translation initiation factor 2 (eIF2α), were detected. 

Taken together, these data suggest that verbascoside protects β-cell from dysfunctions associated with ER-stress and acts by modulating the PERK branch of the UPR, a pathway deregulated in diabetes [41]. To confirm this hypothesis, the cells were treated with tunicamycin, an inhibitor of N-linked glycosylation and a potent ER-stress inducer (Figure 3C). As expected, a seven-hour incubation with 2 µg/mL tunicamycin increased the activation of the PERK pathway (Figure S2) and significantly reduced β-cell viability (Figure 3B). Interestingly, verbascoside pre-treatment (16 µM for 72 h) downregulated the expression of P-eIF2α and partially reverted the action of tunicamycin on β-cell viability. 

Taken together these data strongly support a role of verbascoside in protecting β-cells by attenuation of ER-stress response.

### 3.4. Verbascoside Modulates β-Cells Mitochondrial Activity and Dynamics

**Figure 4:**
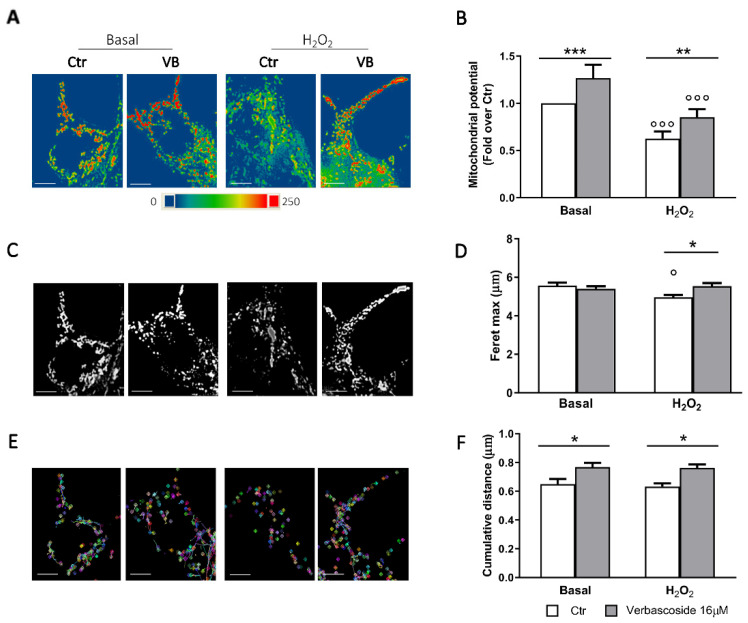
Verbascoside modulates mitochondrial activity, morphology and dynamics. Mouse βtc3 cells were treated with 16 μM verbascoside (VB) for 5 days and then loaded with 100 nM MitoSpy™ Orange CMTMRos. (**A**) Representative images of mitochondria in pseudocolors are shown (blue low intensity, red high intensity). Bar: 5 µm. (**B**) Quantitative analysis of mitochondrial membrane potential measured by fluorimetry (551/576 nm Ex/Em). Data (mean ± SD) were normalized to mitochondrial mass and expressed as fold change over control (n = 4 independent experiments). (Two-way ANOVA post-hoc Tukey’s test °°° *p* < 0.0001 H_2_O_2_ vs. Basal; ** *p* = 0.002, ***p = 0.0002 VB vs. Ctr). (**C**) Representative epifluorescence images of mitochondria are shown. Bar: 5 μm. (**D**) Quantitative analysis of mitochondrial Feret’s maximum diameter (μm); bars illustrate the average responses ± SEM (n = 10-15 cells in three independent experiments). (Two-way ANOVA, post-hoc Tukey’s test. ° *p* = 0.02 H_2_O_2_ vs. Basal; * *p* = 0.02 VB vs. Ctr). (**E**) Video tracking of mitochondrial movements during the 30 s record. Bar: 5 µm. (**F**) Quantitative analyses of mitochondria movements. Bars illustrate the average response (cumulative distance) ± SEM of three independent experiments. (Two-way ANOVA, post-hoc Tukey’s test. * *p* = 0.02 VB vs. Ctr).

MTT viability assays suggest a possible impact of verbascoside directly on mitochondria which ensure the coupling of insulin secretion to the nutritional state and the cell survival in this cell type. Shape and membrane potential are markers of the mitochondrial health [42]. We first evaluated mitochondrial membrane potential by labelling the cells with MitoSpy™ Orange CMTMRos, a permeable dye whose concentration depends on the inner mitochondrial membrane potential, while MitoSpy™ Green FM was used to normalize data to the mitochondrial mass (Figure 4A,B). Membrane potential of mitochondria in verbascoside-treated cells was increased compared to control in both basal and stress conditions. Interestingly, verbascoside metabolites exerted different effects. Caffeic acid did not improve the mitochondrial potential, whereas hydroxytyrosol caused a dose-dependent impairment of mitochondrial function, in agreement with data on cell viability, such an effect was more evident after H_2_O_2_ exposure (Figure S3).

Since function is strictly related to morphology, we measured the Feret’s diameter, area, and circularity of the mitochondria. Acute oxidative stress promotes an extensive mitochondrial fission, thus resulting in smaller, circular mitochondria [42]. Under H_2_O_2_ stress condition, mitochondria of verbascoside-treated cells were more elongated and showed increased surface than control ones (Figure 4C,D and Figure S4). Mitochondrial circularity was lower in verbascoside-treated cells than in controls and differences were more evident in H_2_O_2_ treated cells (Figure S3), again confirming the protective role of the polyphenol. 

The ability of these organelles to modify their shape in response to nutritional states or stressful conditions is under the control of fusion and fission events and requires mitochondrial motility [39]. Tracking of mitochondria movements led us to calculate the cumulative distance travelled and the analysis revealed a significant increase of mitochondria dynamics in the verbascoside treated cells when compared to control, already in basal conditions (Figure 4E,F and Videos S1–S4). The effect was more pronounced after H_2_O_2_ treatment, indeed verbascoside pre-treatment almost completely reverted the impact of oxidative stress on mitochondrial dynamics. 

Taken together these data strongly support a key role of verbascoside in ensuring mitochondria dynamics which is crucial to promote the rapid adaptation of this organelle to stress conditions. 

### 3.5. Verbascoside Impact on Human of Langerhans Survival and Function

**Figure 5:**
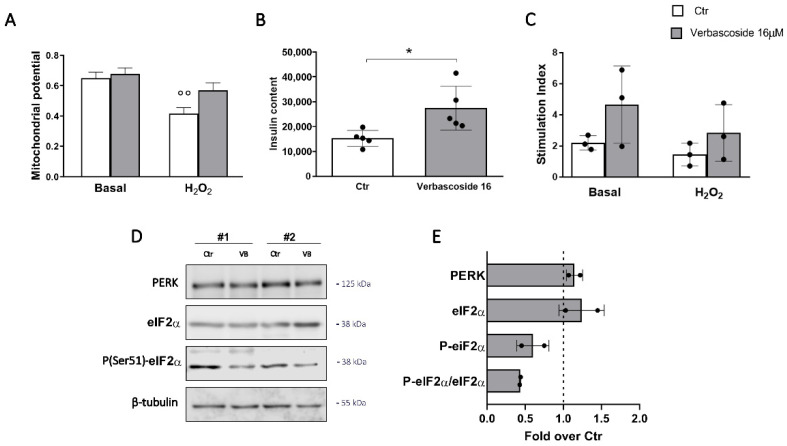
Verbascoside improves human islets of Langerhans function. Islets were incubated with or without 16 µM verbascoside for 5 days. (**A**) Mitochondrial membrane potential. Human islets were loaded with 100 nM MitoSpy™ Orange CMTMRos and 100 μM MitoSpy™ Green FM and the mitochondrial membrane potential and mass were measured by fluorimetry (551/576 nm Ex/Em and 490/516 nm Ex/Em, respectively). The bar graph illustrates the average responses ± SD, data were normalized to the mitochondrial mass (Two-way ANOVA, post-hoc Tukey’s test, °° *p* = 0.0013 H_2_O_2_ vs. Basal). (**B**) The insulin content was evaluated by ELISA assay. Data (mean ± SD) are expressed as mU insulin/g protein (n = 5 independent experiments; Student’s *t*-test, * *p* < 0.05 VB vs. Ctr). (**C**) The insulin release in basal (3.3 mM glucose) and stimulated (16.7 mM glucose) conditions were measured by ELISA assay and data (mean ± SD; n = 3 independent experiments) are expressed as stimulation index (stimulated/basal insulin release). (**D**) Western blot analysis of ER stress markers in islets treated with 16 μM verbascoside (VB) for 5 days (15 μg protein/sample). On the right, the molecular-weight size markers in kDa are reported. (**E**) The quantitative analysis shows a trend toward decrease of P-eIF2α expression and P-eIF2α/eIF2α ratio. Data (mean values ± SD) are expressed as fold change over control (dashed line). (n = 2 different islets isolation, performed in duplicate).

Considering the translational potential of verbascoside application in human health, we verified its impact on human isolated islets of Langerhans, a more relevant model for physiopathological and pharmacological studies on β-cells. Isolated islets were treated with 16 μM verbascoside for 5 days and the mitochondrial membrane potential was evaluated. As reported in Figure 5A, while organelle potential was compromised in control human islets after H_2_O_2_ exposure, the inner membrane potential of mitochondria in verbascoside pre-treated islets did not decrease significantly, supporting the hypothesis of a protective role of verbascoside against ROS-induced β-cell dysfunction. Analysis of ER-stress markers expression showed an important reduction of P-eIF2α fraction in the two distinct islet preparations maintained in 16 µM verbascoside, confirming the downregulation of this pathway also in human samples (Figure 5D). The beneficial effects of the polyphenol treatment were corroborated by the increased insulin content observed in verbascoside-treated islets when compared to controls (Figure 5B). Data dispersion in our experiments did not allow to demonstrate any significant difference of glucose-stimulated insulin secretion (GSIS) in verbascoside-treated islets, although a positive trend seems apparent under both basal and stress conditions (Figure 5C).

## 4. Discussion

Oxidative stress and inflammation are the basis of β-cell dysfunction occurring during T2D [43,44] as such, functional food, nutraceuticals, and phytochemicals have been investigated as tools to prevent type 2 diabetes [45]. In this work, we focused on verbascoside, that is known to exert anti-inflammatory and anti-oxidant activities in neurons [18]. Despite the high structural complexity, verbascoside and isoverbascoside have been found in blood plasma of rats fed with *Lippia citriodora* extract together with some metabolites [20]. In addition, cell accumulation of verbascoside and isoverbascoside has been detected in a breast cancer cell line SKBR3 [23] and studies conducted on Caco-2 cells demonstrate that moderate amounts of verbascoside and isoverbascoside remain intact and bioaccessible after in vitro digestive process. Furthermore, Caco-2 can uptake both molecules through a rapid and linear transport in 10 to 100 μM range [22]. No data have been reported about verbascoside bioavailability in humans, and yet the above-mentioned studies suggest that it may be feasible for non-metabolized verbascoside to cross intestinal barrier, circulate in blood plasma, and exert antioxidant effects on endocrine β-cells of the pancreas. 

We found a dose-dependent (0.8–16 µM) protective role of verbascoside on clonal and human β-cells, both under basal and stress conditions. This effect is due to verbascoside itself and not to its metabolites caffeic acid and hydroxytyrosol, which actually appear cytotoxic at the 16 μM concentration. The concentrations proven active in our experiments are in line with those described in animals fed with verbascoside [20].

Though we do not know whether verbascoside acts extracellularly by binding to a membrane receptor or is internalized by endocytosis and works intracellularly, our data confirm that verbascoside has antioxidant and anti-inflammatory properties, and protects β-cells against ER-stress associated dysfunctions by reducing the UPR and promoting mitochondrial dynamics. 

Indeed, we detected a significant decrease of ROS content in verbascoside treated cells which is further confirmed by the reduction of lipid peroxidation measured through HNE and acrolein expressions. Unlike most of plant polyphenols, verbascoside ROS scavenging activity mainly follows indirect (via upregulation of ROS removing enzymes) rather than direct pathways. In fact, it increases the gene transcription of some antioxidant enzymes through the activation of Nrf2 (NF-E2-related factor 2) pathway and via AhR (aryl hydrocarbon receptor)-dependent mechanism [24,29]. As such, we found increased expression of SOD1 in verbascoside treated cells. 

ROS homeostasis is extremely relevant in β-cells pathology. Due to the shortage of antioxidant enzymes, elevated production of ROS cannot be neutralized and ER stress promoting protein misfolding is induced. ER stress leads to a reduction of insulin transcription and translation through the activation of the UPR [44,46]. This pathway has the main purpose of recovering the ER function through the reprogramming of gene expression, however when overstimulated, it triggers apoptosis. Our data suggest that an important action of verbascoside is to mitigate the UPR, allowing the modifications necessary to repair ER dysfunction, without causing apoptosis. According to this possibility, the expression of BIP, HSP70 and PERK proteins was decreased in verbascoside treated β-cells, and the activation of PERK pathway was attenuated in response to tunicamycin-induced ER stress. A similar action was reported for tyrosol in the insulinoma NIT-1 cell line [47].

Particularly interesting is the ability of verbascoside to modulate the activation of the PERK branch of the UPR. In β-cells this pathway is required to maintain the basal secretory homeostasis and β-cell survival, and it is severely deregulated in diabetes [41,48]. Even more intriguing, common variants at PERK contribute to the risk of prediabetes and recessive mutations in the EIF2AK3 gene (encoding PERK) underlie susceptibility to the Wolcott–Rallison syndrome characterized by permanent neonatal insulin dependent diabetes [49,50]. 

Downregulation of the IκBα-NFκB inflammatory pathway was also detected in our system in the presence of verbascoside. NFκB represents the main inflammatory pathway in β-cells and its sustained activation initiates a cascade of events culminating in β-cell death. In stressed β-cells, the reduction of NFκB expression protects pancreatic β-cells from diabetogenic agents [27,51], further supporting the verbascoside application in diabetes prevention. Interestingly, PERK signaling activates the transcription factors NFR2 (implicated in the redox homeostasis) and NFκB, thus explaining the anti-oxidant and anti-inflammatory effects of verbascoside [40,52]. 

Our data also reveal an important role of verbascoside on mitochondrial activity and dynamics. Marker of mitochondrial operational quality is their dynamic, a process characterized by coordinated cycles of fusion and fission events that regulates mitochondrial number, distribution, morphology and their membrane potential [39]. In line with this possibility, verbascoside improves βtc3 mitochondrial membrane potential both under basal and stress conditions. Again, the effect is due to verbascoside and not to its metabolites, as caffeic acid does not improve mitochondrial function and hydroxytyrosol significantly decreases the organelle potential. Increased mitochondrial dynamics and changes in their morphology were observed by tracking mitochondrial movements, thus suggesting that verbascoside promotes a mito-morphosis program. Reshaping of the cellular mitochondrial network affects the assembly of the respiratory chain super-complexes, thus altering not only the cell metabolism but also its redox state and enabling cells to better counteract oxidative stress and inflammation [5,37,39]. 

Although the molecular mechanism is still unknown, we can hypothesize that verbascoside, as other polyphenols, protects mitochondrial DNA by reducing ROS concentration [53] and prevents the opening of mitochondrial permeability transition pore [54], ensuring the physiological mitochondrial activity. Another intriguing possibility is that modification of mitochondria dynamics and activity is again mediated by the action of verbascoside on the UPR. Indeed, a recent research in the mitochondrial field indicates that sites of ER-mitochondria interaction play a key role in the control of mitochondrial dynamics and activity in response to oxidative stress and PERK is involved in the phenomenon [55].

Most in vitro studies with polyphenols were performed in cell lines, often of tumoral origin, which differ from original cells in terms of metabolism and ROS production. We here provide evidence that verbascoside exerts a protective effect also on human isolated islets of Langerhans, the final target of intervention in diabetes. Interestingly, the polyphenol mitigates the PERK signaling activation already under basal conditions, thus increasing the insulin content and preventing oxidative stress-mediated mitochondrial dysfunctions. It remains to be elucidated whether the polyphenol effect on insulin content reflects the compound ability to prevent β-cell death, to mitigate ER stress or to directly control the insulin gene expression, as shown for other polyphenolic compounds [56]. These data are particularly relevant considering the possible use of verbascoside as a complementary therapy in diabetes treatment.

## 5. Conclusions

The in vitro studies reported here on clonal and human β-cells, indicate that verbascoside exerts protective effects against ER-stress associated dysfunctions, mitigating the activation of the PERK branch of the UPR and improving mitochondria dynamics. As disruption of ER homeostasis triggers β-cells damage and diabetes, these data provide a rationale for the possible use of verbascoside as nutraceutical in disease prevention and treatment. Yet, many issues need to be resolved for an effective clinical application of verbascoside. Despite the wealth of laboratory studies, reliable clinical studies confirming the health effects of verbascoside in vivo are limited. Furthermore, increasing its stability and bioavailability is mandatory for the future application of this compound in human health. From this point, verbascoside is an interesting molecule because its scaffold has different reactive sites that can be modified by combinatory chemistry. We expect that the information resulting from these studies will open avenues for therapeutic modulation of oxidative stress and inflammation in pathological condition by using natural compounds.

## Supplementary Materials

The following are available online at https://www.mdpi.com/2227-9059/8/12/582/s1. Figure S1: Effects of caffeic acid and hydroxytyrosol on β-cell viability. Figure S2: Effects of verbascoside on tunicamycin-induced ER stress. Figure S3: Effects of caffeic acid and hydroxytyrosol on mitochondrial membrane potential. Figure S4: Quantitative image analyses of mitochondria in cells treated with caffeic acid and hydroxytyrosol. Video S1,S2 Videos: Mitochondria dynamics recorded under basal conditions, in the absence (S1) or presence (S2) of verbascoside. Video S3, S4: Mitochondria dynamics recorded under oxidative stress conditions, in the absence (S3) or presence (S4) of verbascoside.
