# Supplementary material for: Verbascoside Protects Pancreatic β-Cells against ER-Stress
Source: Biomedicines. 2020 Dec 8;8(12):582. doi: 10.3390/biomedicines8120582 (PMC7762434; doi:10.3390/biomedicines8120582)
Supplement: Supplementary file 1 [file biomedicines-08-00582-s001.zip › biomedicines-1021518-Supplementary figures.docx]

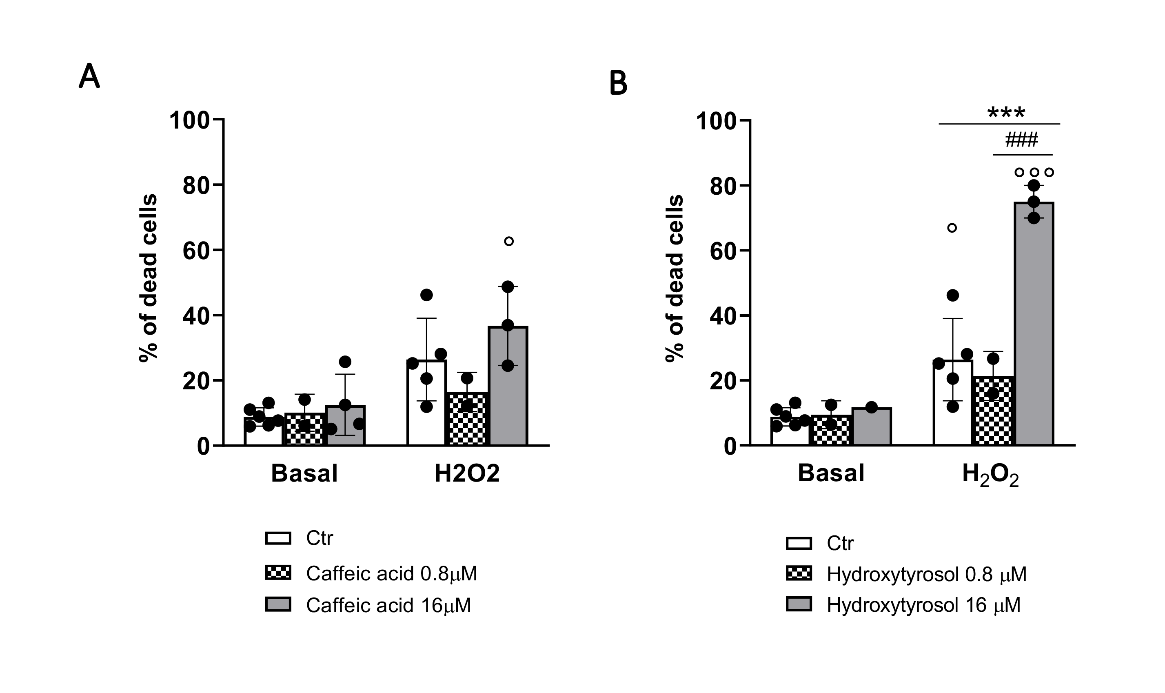


**Figure S1. Effects of caffeic acid and hydroxytyrosol on β-cell viability**. Mouse βtc3 cells were treated with caffeic acid (A) and hydroxytyrosol (B) (0.8-16 μM) for 5 days, and the percentage of dead cells was analysed by flow cytometry. Ethanol treated cells were used as controls. Dead cells (mean values ± SD) are expressed as percentage of total cells; experiments were performed in triplicate. (Two-way ANOVA, post-hoc Tukey’s test*.* Hydroxytyrosol: ° p = 0.02, °°° p = 0.0007 H_2_O_2_ vs basal; *** p < 0.0001 hydroxytyrosol 16 µM vs Ctr; ### p = 0.004 hydroxytyrosol 16 µM vs 0.8 µM) (Two-way ANOVA, post-hoc Tukey’s test*.* Caffeic acid° p = 0.034, °°° p = 0.004 H_2_O_2_ vs basal).


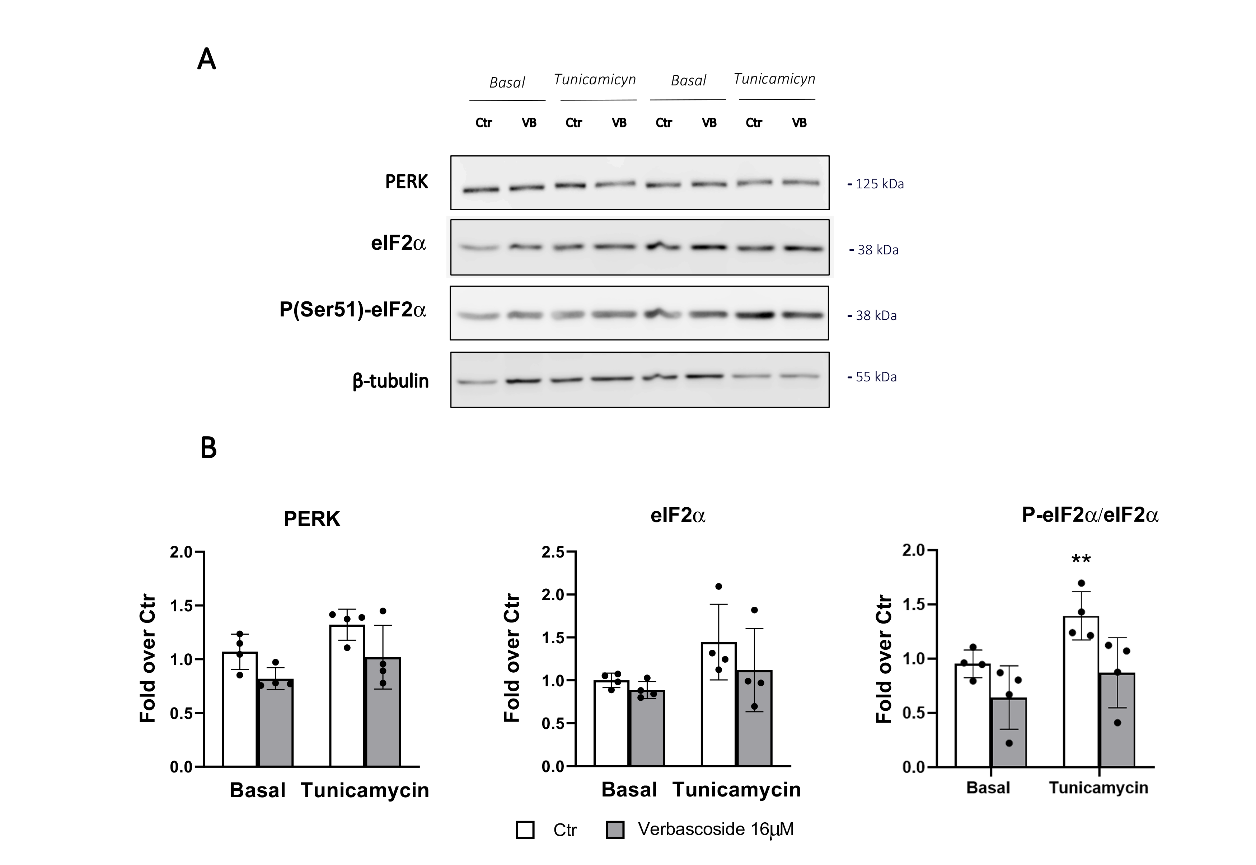


**Figure S2. Effects of verbascoside on tunicamycin-induced ER stress.** Mouse βtc3 cells were incubated with 16 μM verbascoside (VB) for 5 days and ER stress was induced by 2 μg/mL tunicamycin treatment for 7 hours. **A)** Representative western blot images of ER stress markers (30 μg protein/sample). On the right, the molecular-weight size markers in kDa are reported. **B)** Quantitative analysis of PERK, eIF2α and P-eIF2α expressions. Bars illustrate the average response ± SD of four independent experiments (Two-way ANOVA post-hoc Tukey’s test*.* P = 0.006 Ctr, tunicamycin vs basal).


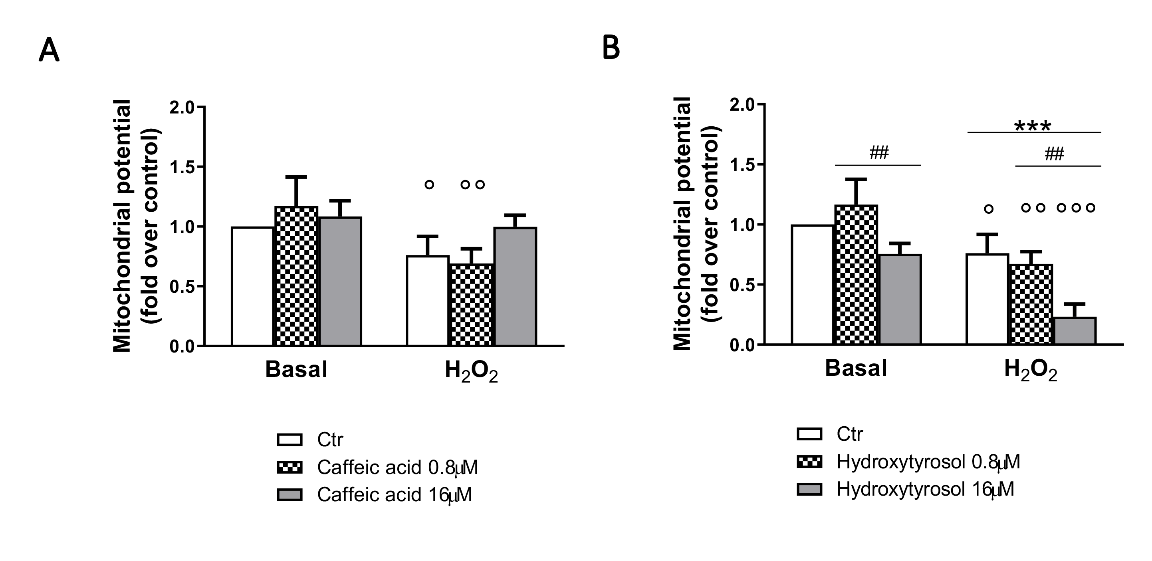


**Figure S3. Effects of caffeic acid and hydroxytyrosol on mitochondrial membrane potential**. Mouse βtc3 cells were treated with caffeic acid (A) and hydroxytyrosol (B) (0.8-16 μM) for 5 days, and then loaded with MitoSpy™ Orange CMTMRos. The mitochondrial membrane potential was measured by fluorimetry (551/576 nm Ex/Em) and data (mean ± SD) are normalized to mitochondrial mass and expressed as fold change over control (n=4 independent experiments). (Two-way ANOVA, post-hoc Tukey’s test. Caffeic Acid:° p = 0.034, °° p = 0.004 H_2_O_2_ vs basal) (Two-way ANOVA, post-hoc Tukey’s test hydroxytyrosol: °p = 0.019; °°p = 0.002; °°° p = 0.0007 *H_2_O_2_* vs basal; *** p < 0.0001 hydroxytyrosol 16 µM vs Ctr; ## p = 0.008 hydroxytyrosol 16 µM vs 0.8 µM).


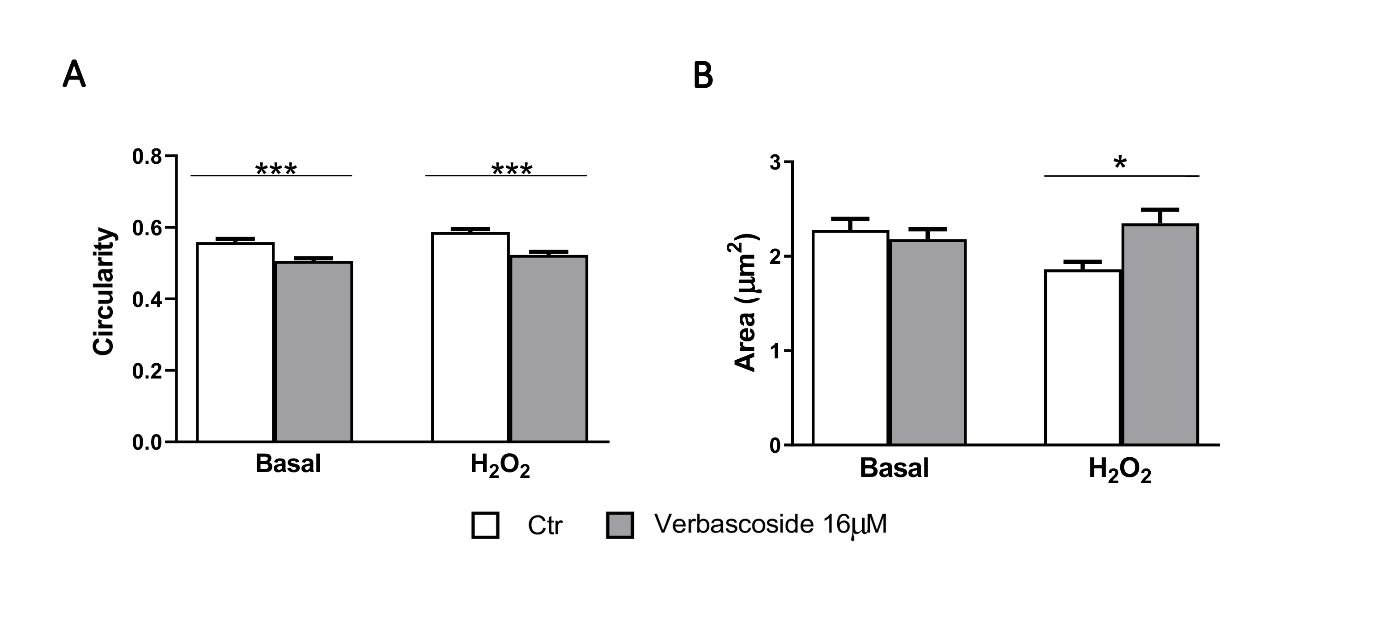


**Figure S4. Quantitative image analyses of mitochondria in cells treated with verbascoside.** Mouse βtc3 cells were treated with 16 μM verbascoside (VB) for 5 days and mitochondria were labeled with MitoSpy™ Orange CMTMRos and imaged. A) Circularity ($\frac{4\pi{Area}^{2}}{{Perimeter}^{2}}$) and B) area (µm^2^) were evaluated by quantitative analysis. Bars illustrate the average responses ± SD (N = 10-15 cells in three independent experiment). (Two-way ANOVA, post-hoc Tukey’s test * p = 0.012, *** p < 0.0001 VB vs Ctr).
