# Supplementary material for: Verbascoside Protects Pancreatic β-Cells against ER-Stress
Source: Biomedicines. 2020 Dec 8;8(12):582. doi: 10.3390/biomedicines8120582 (PMC7762434; doi:10.3390/biomedicines8120582)
Supplement: Supplementary file 1 [file biomedicines-08-00582-s001.zip › Legend of Supplementary videos S1-S4.docx]

**Supplementary videos V1-V4 legend**

**Supplementary video V1. Mitochondria dynamics recorded under basal conditions.** Live fluorescence cell imaging of mitochondria movements over a period of 30 sec, in cells labelled with MitoSpy™ Orange CMTMRos (Sampling frequency: 1Hrz)

**Supplementary video SV2. Mitochondria dynamics recorded under basal conditions in verbascoside treated cells.** Live fluorescence cell imaging of mitochondria movements over a period of 30 sec, in cells pretreated with 16 µM verbascoside for five days. (Sampling frequency: 1Hrz)

**Supplementary video SV3. Mitochondria dynamics recorded under oxidative stress conditions.** Live fluorescence cell imaging of mitochondria movements over a period of 30 sec, in cells exposed to oxidative stress (500 μM H_2_O_2_ for 20 min) (Sampling frequency: 1Hrz).

**Supplementary video SV4. Mitochondria dynamics recorded under oxidative stress conditions in verbascoside treated cells.** Live fluorescence cell imaging of mitochondria movements over a period of 30 sec, in cells pretreated with 16 µM verbascoside for five days and exposed to oxidative stress (500 μM H_2_O_2_ for 20 min) (Sampling frequency: 1Hrz).
